# A Three-Dose mRNA COVID-19 Vaccine Regime Produces Both Suitable Immunogenicity and Satisfactory Efficacy in Patients with Solid Cancers

**DOI:** 10.3390/vaccines11061017

**Published:** 2023-05-23

**Authors:** Urska Janzic, Urska Bidovec-Stojkovic, Peter Korosec, Katja Mohorcic, Loredana Mrak, Marina Caks, Maja Ravnik, Erik Skof, Matija Rijavec

**Affiliations:** 1 Department of Medical Oncology, University Clinic of Respiratory and Allergic Diseases Golnik, 4204 Golnik, Slovenia; katja.mohorcic@klinika-golnik.si (K.M.); loredana.mrak@klinika-golnik.si (L.M.); 2 Medical Faculty Ljubljana, University of Ljubljana, 1000 Ljubljana, Slovenia; eskof@onko-i.si; 3 Laboratory for Clinical Immunology and Molecular Genetics, University Clinic of Respiratory and Allergic Diseases Golnik, 4204 Golnik, Slovenia; urska.bidovec-stojkovic@klinika-golnik.si (U.B.-S.); peter.korosec@klinika-golnik.si (P.K.); matija.rijavec@klinika-golnik.si (M.R.); 4 Faculty of Pharmacy, University of Ljubljana, 1000 Ljubljana, Slovenia; 5 Department of Oncology, University Medical Centre Maribor, 2000 Maribor, Slovenia; marina.caks@ukc-mb.si (M.C.); maja.ravnik@ukc-mb.si (M.R.); 6 Department of Medical Oncology, Institute of Oncology Ljubljana, 1000 Ljubljana, Slovenia; 7 Biotechnical Faculty, University of Ljubljana, 1000 Ljubljana, Slovenia

**Keywords:** solid cancer, COVID-19 vaccination, booster third dose, immunogenicity

## Abstract

Background: The recommended booster third dose of vaccination against COVID-19 in cancer patients seems reasonable to protect them against a severe disease course. A prospective study was designed to assess the immunogenicity, efficacy, and safety of COVID-19 vaccination in this cohort. Methods: Patients with solid malignancies on active treatment were followed up after the primary course and booster third dose of vaccination to assess their anti-SARS-CoV-2 S1 IgG levels, efficacy in the case of SARS-CoV-2 infection, and safety. Results: Out of 125 patients receiving the primary course of vaccination, 66 patients received a booster third dose of mRNA vaccine, with a 20-fold increase in median anti-SARS-CoV-2 S1 IgG levels compared to Ab levels six months post-primary course of vaccination (*p* < 0.0001). After the booster third dose, anti-SARS-CoV-2 S1 IgG levels were comparable to healthy controls (*p* = 0.113). There was a decline in Ab levels 3 (*p* = 0.0003) and 6 months (*p* < 0.0001) post-third booster dose. No patients had either a severe disease course or a lethal outcome in the case of SARS-CoV-2 infection after the third booster dose. Conclusion: The third booster vaccination dose against COVID-19 in solid cancer patients triggers substantial immunogenicity and is safe and effective for preventing a severe COVID-19 disease course.

## 1. Introduction

The recent COVID-19 pandemic has dramatically changed the world in which we live and left devastating consequences, with more than 680 million people infected and over 6.8 million deaths worldwide since its beginning in January 2020. Only in Slovenia, with a population of 2.1 million, there have been over 1.3 million confirmed infections and over 7000 deaths from COVID-19 alone in these 3 years [1]. Cancer patients were extremely vulnerable at this time due to their illness, treatment modalities, and frequent visits to healthcare facilities, making them easy targets for contracting the virus. Sadly, the death toll in this population reached a devastating 25–33% in cases of COVID-19 infections [2,3,4].

A rapid global effort to invent and produce vaccines against COVID-19 has produced results in less than a year’s time. However, none of these vaccines were tested in terms of efficacy, immunogenicity, or safety in special, immunocompromised populations such as cancer patients [5,6]. Since vaccine efficacy or safety is not always optimal in patients diagnosed with or treated for cancer, there was a gap in the knowledge for the vaccination of this patient population. Many national and international teams joined forces and started cohort observational studies for cancer patients vaccinated against COVID-19 [7,8,9,10,11]. As shown by us and others, the anxiously awaited COVID-19 vaccines showed adequate immunogenicity, efficacy, and safety in patients with solid malignancies after the primary course of vaccination [8,11,12,13,14]. However, the immunogenicity and efficacy varied greatly across cancer patient populations and were mainly dependent upon the type of cancer (patients with haematological cancers yielded much lower seroconversion rates than those with solid cancers) or type of therapy received (again, patients receiving therapies directed against parts or whole lymphocytes were less likely not to produce effective antibodies against COVID-19). Moreover, the type of vaccine received was also an important factor in the immune responses, with mRNA vaccines producing higher and more potent seroconversion rates in comparison with vector vaccines [15,16,17].

Concerning the effect of the primary course of vaccination waned 3–6 months afterward, and the virus was persistent and evasive, with new and dangerous variants of concern emerging at a rapid pace, the CDC was stimulated to approve and recommend the third booster dose of the COVID-19 vaccine, especially in patients with impaired immune systems [18].

Our prospective observational cohort study (PRO-ONCO-COVID-19) started in early 2021 when Slovenia gained access to COVID-19 vaccines, and cancer patients were one of the priority groups receiving them. We have already reported the immunogenicity and safety of COVID-19 vaccination after our population’s primary course of vaccination [14]. Here, we report an update on our patient population that also received the third booster vaccine and was followed for six months thereafter for antibody (Ab) response assessment.

## 2. Materials and Methods

### 2.1. Study Design and Participants

A prospective observational cohort study of patients with solid cancers receiving systemic cancer therapy at the moment of vaccination or in the past year before vaccination against COVID-19 was conducted. All patients gave written informed consent before study inclusion. The study and its amendment for following patients after the third booster dose of vaccine was approved by the Slovenian National Ethics Committee (No. 0120-39/2021/6 and 0120-39/2021/9). This trial was conducted in accordance with the ethical principles of the Declaration of Helsinki. 

### 2.2. Procedures

We have already described procedures in detail in our first publication [14]. In short, patients were treated oncologically as per standard of care at two academic Slovenian oncology centres and vaccinated with either mRNA vaccines BNT162b2 (Pfizer/BioNTech) or mRNA-1273 (Moderna) or adenoviral AZD1222 (AstraZeneca) vaccines per manufacturing instructions for the primary course of vaccination. All of the patients received one of the mRNA vaccines for the third booster dose of vaccination as per National Vaccination Strategy recommendations about six months after the primary course of vaccination. Prior COVID-19 infection was not an exclusion factor but was documented, as were all of the cases of COVID-19 infection that occurred during the observation period. In addition, the severity of COVID-19 infection was assessed in case of infection. The patients were also asked to report moderate or severe adverse effects that might appear after receiving the third booster dose. 

Blood samples for anti-SARS-CoV-2 IgG determination from patients were obtained at prespecified time points: time point 0: before the vaccination; time point 1: up to 3 weeks after the first vaccination dose; time point 2: up to 3 weeks after the second vaccination dose; time point 3: 3 months after the complete primary course of vaccination; time point 4: 6 months after the primary course of vaccination; time point 5: up to 4 weeks after the third booster dose; time point 6: 3 months after the third booster dose; time point 7: 6 months after the third booster dose; and time point 8: in case patients were not vaccinated with third booster dose and they complied to be followed for up to 12 months after the primary course of vaccination. Results were compared to anti-SARS-CoV-2 IgG antibodies produced by a cohort of healthy volunteers with blood samples that were also obtained after the third booster dose of vaccine was administered. 

### 2.3. Anti-SARS-CoV-2 S1 IgG Antibodies Detection in Serum

According to the manufacturer’s instructions, the immunogenicity was assessed by S1-protein-based commercial ELISA assay IDK® anti-SARS-CoV-2 IgG by Immundiagnostik AG (Bensheim, Germany). The assay detects IgG antibodies directed against the receptor-binding domain of the S1 subunit of the spike protein of SARS-CoV-2. It has no cross-reactivity to plasma probes for Adenovirus, Epstein–Barr Virus, Influenza A/B, HCoV-229E, HCoV-HKU1, HCoV-NL63, and HCoV-OC4. The results show an excellent correlation with the WHO International Standard for anti-SARS-CoV-2 immunoglobulin measurement (R2 = 0.9909; NIBSC code: 20/136) and a strong correlation with SARS-CoV-2 neutralization [19]. Since there was a great need to harmonize all of the studies that reported immunogenicity and antibody production after COVID-19 vaccination, a proposition paper by Barriere et al. [20] suggested the use of binding antibody units (BAU/mL) for reporting across all trials. Our results were primarily in ng/mL, and to obtain the concentration in BAU/mL, the results were divided by a factor of 20 (20 ng/mL ≙ 1 BAU/mL), according to manufacturer’s guidelines. [21] Measurements over 5000 BAU/mL were truncated at 5000 BAU/mL (the upper limit of the assay without further dilutions). Subjects with values < 40 BAU/mL were considered non-responders, those with values between 40 and 260 BAU/mL were considered low-responders, and subjects with values ≥ 260 BAU/mL were considered to have an appropriate immune response to vaccination [20].

### 2.4. Statistical Analysis and Outcomes

Data distribution was evaluated using the D’Agostino and Pearson test. Wilcoxon signed-rank test and Mann–Whitney test were used as appropriate to calculate differences of anti-SARS-CoV-2 S1 IgG antibody levels between different time points and groups. The frequency distribution of patients with anti-SARS-CoV-2 S1 IgG antibodies between different time points and groups was compared with Fisher’s exact test based on contingency tables. Statistical analysis was performed using GraphPad PRISM software (version 9.2 for Windows; GraphPad Software, San Diego, CA, USA). A *p*-value of less than 0.05 was considered statistically significant.

We primarily investigated the immunogenicity of COVID-19 vaccines with anti-SARS-CoV-2 S1 IgG antibodies produced after vaccination of cancer patients with approved and available COVID-19 vaccines in Slovenia after the first, second, and third booster doses of vaccination. Antibodies were also monitored later at three and six months to assess the endurance of antibody protection and the dynamics of alteration. 

As a secondary endpoint, we examined the proportion of patients with SARS-CoV-2 infection after the complete primary course of vaccination who did not agree to be vaccinated further and compared them to the group that was vaccinated with the third booster dose. In addition, we looked at the disease outcomes of patients that were vaccinated with three doses and were infected with SARS-CoV-2 afterward. The course of the disease was correlated with the WHO criteria of mild (symptomatic patients meeting the definition for COVID-19 without evidence of viral pneumonia or hypoxia), moderate (clinical signs of pneumonia—fever, cough, dyspnoea, fast breathing, but no signs of severe pneumonia, including SpO2 ≥ 90% on room air), or severe disease course (clinical signs of pneumonia—fever, cough, dyspnoea, plus one of the following: respiratory rate > 30 breaths/min; severe respiratory distress; or SpO2 < 90% on room air) [22].

## 3. Results

Between March and July 2021, 125 patients with a known solid malignancy treated at two academic institutions in Slovenia who were also vaccinated, were included and followed thereafter until December 2022 (six months after the last patient received the booster third dose of vaccine).

**Figure 1:**
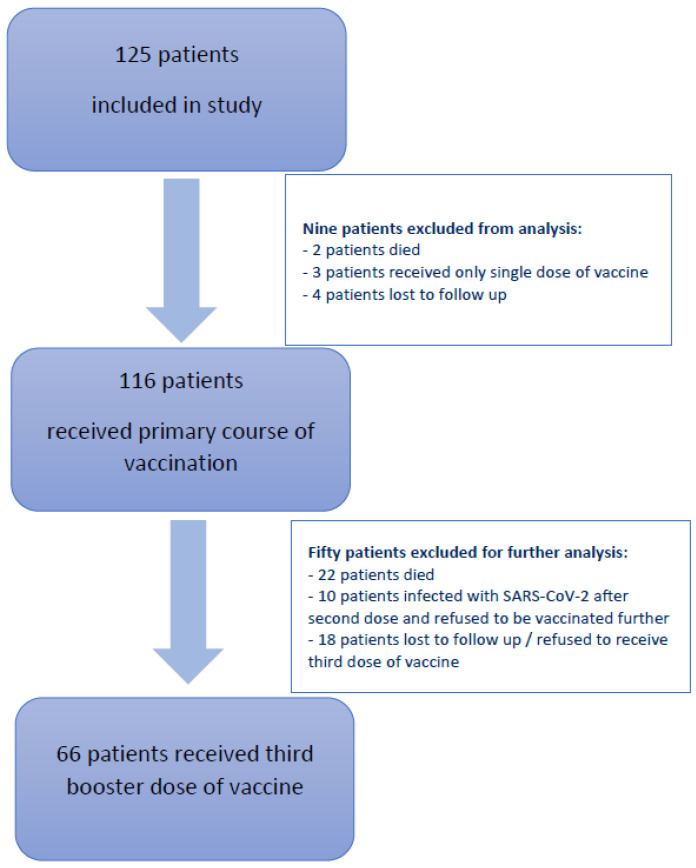
Consort diagram depicting patients included in our study and reasons for the exclusion from further analysis.

At the time of the first analysis, nine patients were excluded from the primary analysis: two patients died, three patients received only a single vaccine dose instead of the recommended two, and four patients were lost to follow-up or did not adhere to the study protocol. Of the 116 patients that received the entire primary course of vaccination, 66 patients also received the third booster dose, which is presented in detail in this analysis. Data from the remaining 50 patients that were excluded from the analysis are presented in the consort diagram in Figure 1. 

Additionally, 10 patients got infected with SARS-CoV-2 after the primary course of vaccination and refused to be vaccinated further, but they adhered to the original protocol and were followed up until 12 months after the primary course of vaccination. 

The median age of cancer patients receiving the booster third dose was 64 years (range 43–83 years): 59% were female, 80% were being treated for NSCLC (non-small cell lung cancer), and most of the patients (70%) were treated in the metastatic setting. The majority of patients (91% and 97%) received the mRNA BNT162b2 vaccine for the primary course and booster third dose of vaccination as well. More details on the patients’ characteristics are presented in Table 1.

### 3.1. Humoral Immune Response to COVID-19 Booster Third Vaccination

Right before the booster third dose of vaccination (time point 5: six months after the primary course of vaccination), 12 (18%) of the patients had negative anti-SARS-CoV-2 S1 IgG antibodies with levels below 40 BAU/mL, and another 30 (45%) of patients had low detectable anti-SARS-CoV-2 S1 IgG antibodies with levels between 40 and 260 BAU/mL. The median time between the second and third booster doses was 216 days (range 153–300 days), which corresponds well to the proposed six-month period between the two doses. All of the patients received mRNA-based third booster doses of the vaccine, and the levels of their antibodies rose significantly after receiving this vaccination course. After the third booster shot, only one patient remained with negative anti-SARS-CoV-2 S1 IgG antibodies, and two were low responders with anti-SARS-CoV-2 S1 IgG antibodies, with levels of 100 BAU/mL and 223 BAU/mL, respectively. The remaining patients were good responders, with Ab levels above 260 BAU/mL, and the whole group attained median (range) titers of 3141 BAU/mL (0–5000 BAU/mL). Off note, four patients that received the adenoviral vaccine for the primary course of vaccination, also responded well to the booster third dose of the mRNA vaccine with a median anti-SARS-CoV-2 S1 IgG Ab titer of 2111 BAU/mL (range 223–5000 BAU/mL). The median anti-SARS-CoV-2 S1 IgG Ab titer value after the complete primary course of vaccination was 993 BAU/mL (0–5000 BAU/mL) and dropped to 143 BAU/mL (0–5000 BAU/mL) six months later, which means that over 3-fold and 20-fold increases in the levels of anti-SARS-CoV-2 S1 IgG Ab after the booster third dose of the vaccination were achieved, with both differences being statistically significant (*p* < 0.0001). 

Similarly, out of 42 healthy controls tested after the third booster dose of the vaccine, they reached a median anti-SARS-CoV-2 S1 IgG Ab level of 3903 BAU/mL (376–5000 BAU/mL), which was only slightly higher than our cancer patient cohort and was not statistically significant (*p* = 0.113).

**Figure 2:**
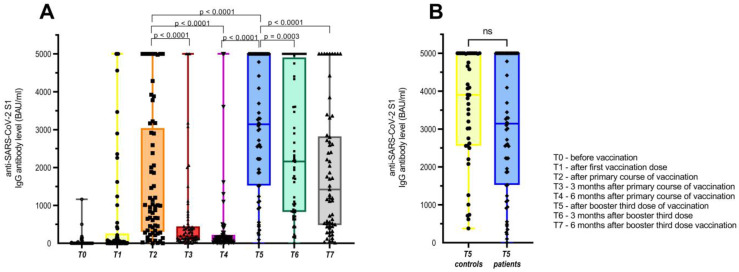
(**A**) Levels of anti-SARS-CoV-2 S1 IgG Ab for the whole group of patients that received booster third dose of COVID-19 vaccine and (**B**) comparison with anti-SARS-CoV-2 S1 IgG Ab of healthy controls after third booster dose.

The anti-SARS-CoV-2 S1 IgG Ab levels declined again, three and six months after the third booster dose, with significantly lower median levels of 2162 BAU/mL (*p* = 0.0003) and 1421 BAU/mL (*p* < 0.0001), respectively. However, the decline was not as steep as seen after the primary vaccination course, with median anti-SARS-CoV-2 S1 IgG Ab values of 188 and 143 BAU/mL (both *p* < 0.0001), respectively. Interestingly, 6 months after the booster third dose of the vaccination, the IgG titers were comparable to those after the complete primary course of vaccination and were 10-fold higher than 6 months after the primary vaccination course. The distribution of anti-SARS-CoV-2 S1 IgG Ab values in cancer patients and the comparison with healthy controls are presented in Figure 2A,B. 

**Figure 3:**
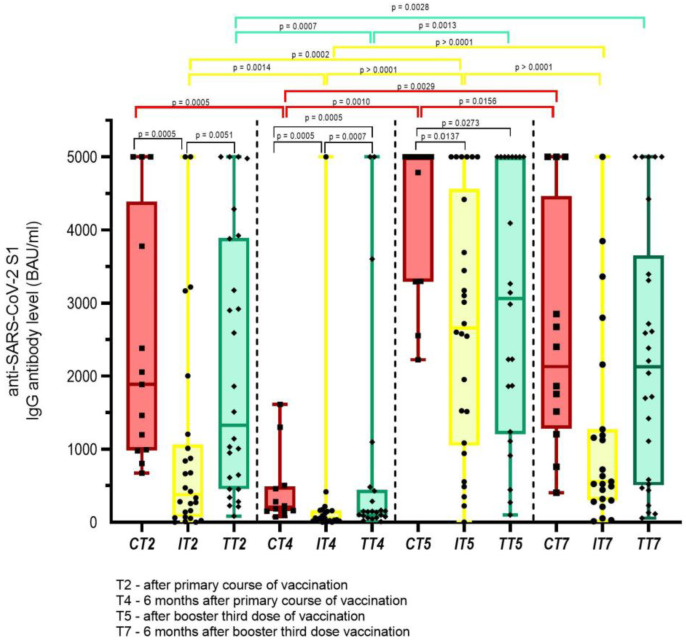
Anti-SARS-CoV-2 S1 IgG Ab levels according to anticancer therapies at different time points; red bars—chemotherapy (C); yellow bars—immunotherapy with immune checkpoint inhibitors (I); green bars—targeted therapy (T). Only statistically significant differences are depicted in Figure.

Again, some differences according to anticancer therapies were noted regarding anti-SARS-CoV-2 S1 IgG Ab production, as shown in Figure 3. There was a significant drop in the levels of anti-SARS-CoV-2 S1 IgG Ab six months after the primary course of vaccination across all subgroups of anticancer therapies, namely chemotherapy, immunotherapy with ICI, and targeted therapy, with the median antibody values dropping by 9.2 fold (*p* = 0.0005), 6.6 fold (*p* = 0.001), and 9.4 fold (*p* = 0.0007), respectively. Conversely, there was a significant rise in the median levels of anti-SARS-CoV-2 S1 IgG Ab after receiving the third booster dose of the vaccine from 203 BAU/mL to 5000 BAU/mL (*p* = 0.001), 57 BAU/mL to 2659 BAU/mL (*p* < 0.0001), and from 140 BAU/mL to 3062 BAU/mL (*p* = 0.001) for patients receiving chemotherapy, immunotherapy with ICI, and targeted therapy, respectively. Again, a drop in the levels of anti-SARS-CoV-2 S1 IgG Ab was noted six months after the third booster dose, which was statistically significant for both patients receiving immunotherapy with ICI by 4.7 fold (*p* < 0.0001), and for patients receiving chemotherapy by 2.3 fold (*p* = 0.016), but not for patients on targeted therapy—who dropped by 1.4 fold (*p* = 0.154). It should be noted, however, that 10/13 patients receiving chemotherapy had stopped with cytotoxic drugs by the time they reached the booster third vaccination dose. 

### 3.2. Safety and Humoral Immune Response of Cancer Patients Vaccinated with Three Doses of COVID-19 Vaccine and Those Infected with SARS-CoV-2 after the Primary Course of Vaccination

**Figure 4:**
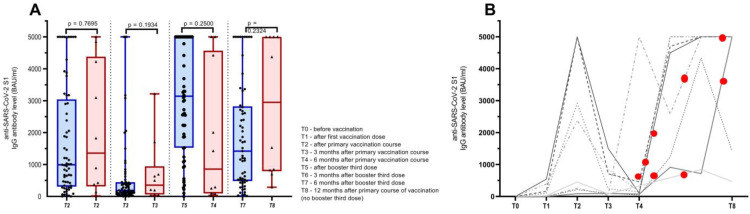
(**A**) Antibody (Ab) titers of cancer patients receiving primary course of vaccination and booster third dose (blue bars) and patients receiving primary course of vaccination and being infected with SARS-CoV-2 after the primary course of vaccination (red bars). (**B**) Schematic representation of anti-SARS-CoV-2 S1 IgG Ab dynamics in patients that were infected with SARS-CoV-2 after primary course of vaccination (each line representing an individual patient) with red dots demonstrating time of infection according to vaccination status.

As discussed above, there were 66 patients that received three doses of the COVID-19 vaccine and 10 patients that refused to be vaccinated three times because of a recent COVID-19 infection after the primary course of vaccination but continued to provide blood samples as per the first study protocol, which was 3, 6, and 12 months after the primary course of vaccination. The SARS-CoV-2 infection commenced at a median of 208 days after the primary course of infection (range 133–361 days). Eight patients had mild disease courses, and two patients had a moderate disease course and were applied required antiviral medication and synthetic antibodies, respectively. Of note, the two patients with a moderate COVID-19 disease course became infected 202 and 248 days after the primary course of vaccination, respectively. As for the anti-SARS-CoV-2 S1 IgG Ab levels, there was no significant difference between the corresponding time points of patients receiving the third booster dose of the vaccine and the group of patients infected after the primary course of vaccination, median (range) anti-SARS-CoV-2 S1 IgG Ab titers 3141 (0–5000) BAU/mL and 857 (31–5000) BAU/mL (*p* = 0.250), respectively. Additionally, six months after the booster third dose and the corresponding infected patients 12 months after the primary course of vaccination, the median (range) anti-SARS-CoV-2 S1 IgG Ab levels were 1911 BAU/mL and 2949 (289–5000) BAU/mL (*p* = 0.232). More details are presented in Figure 4. 

There were no new safety concerns after the third booster dose vaccination. Patients were only asked to report moderate or severe adverse events that would interfere with their daily activities or if they needed medical assistance after the third vaccination, and there were no such reports. Most patients only complained of mild pain at the injection site or feeling feverish with no consequences to their daily activities. 

## 4. Discussion

Our prospective longitudinal study validates the sensible decision of health authorities to vaccinate cancer and other immunocompromised patients with a booster third dose of the mRNA COVID-19 vaccine in order to protect them from harmful consequences in the case of a SARS-CoV-2 infection. As shown in this study, patients with solid malignancies mount a remarkable amount of anti-SARS-CoV-2 S1 IgG Ab after the booster third dose of the vaccine, which is especially pronounced when comparing the low anti-SARS-CoV-2 S1 IgG Ab levels six months after the primary course of vaccination [13,14,23,24]. Most of our patients were vaccinated in the 6-month frame post-primary course of vaccination, similar to other studies reported thus far, and confirmed the significant rise of anti-SARS-CoV-2 S1 IgG Ab levels compared to 3 and 6 months after the primary course of vaccination [23,24,25,26,27,28,29,30,31].

Afterward, a decrease in the absolute number of anti-SARS-CoV-2 S1 IgG Ab titres was observed 3 and 6 months after the third booster vaccine dose, which was also statistically significant, and comparable to two other studies [32,33]. Nevertheless, the absolute levels of the anti-SARS-CoV-2 S1 IgG Ab levels 6 months after the booster third vaccination dose were comparable to those after the complete primary course of vaccination and 10-fold higher than 6 months after the primary vaccination course. Data about the duration of the immune response and the level of anti-SARS-CoV-2 S1 IgG Ab levels after the booster’s third dose are crucial for informed decision-making and further planning of vaccine administration in case of need. Additionally, assessing T-cell immunity or the neutralization capacity of VOC active at the time of infection spread is equally important for informed decision-making [20,33].

Concerning the type of anticancer therapy received, our study showed differences in anti-SARS-CoV-2 S1 IgG Ab levels after the third booster dose, with patients on chemotherapy having the highest anti-SARS-CoV-2 S1 IgG Ab levels, followed by patients on targeted therapy and immunotherapy with immune checkpoint inhibitors. Afterward, the Ab level steeply dropped in patients receiving chemotherapy and immunotherapy than those on targeted therapy, but the difference was not significant in patients receiving targeted therapy. It seems that patients treated with immune checkpoint inhibitors (ICIs) produce lower Ab levels than patients on chemo- or targeted therapy. Since ICIs are showing an immunomodulatory mode of action in patients infected with SARS-CoV-2, thus reducing cytotoxic effects and cytokine release, there could also be a potential effect on delaying anti-SARS-CoV-2 S1 IgG antibody production after vaccination. Similarly, no differences in Ab levels were also noticed in an Italian group of patients, regardless of the anticancer treatment received [29]. Only one patient remained seronegative, and two were low responders in our patient cohort. Other researchers have produced comparable results, with only sporadic patients failing to produce adequate anti-SARS-CoV-2 S1 IgG Ab levels that are deemed protective [25,26,30]. The reason for this is unclear, but probably lies in impaired immune responses, either due to the disease itself or the anticancer treatment received. For instance, patients with hematologic malignancies fail to produce adequate immunogenicity or even seroconversion after the same vaccination schedule, which is partly due to impaired lymphocyte function and partly due to the lymphocyte inhibitor therapies received [34,35].

In our study, no significant differences in the anti-SARS-CoV-2 S1 IgG Ab levels after the third booster dose comparing the cohort of solid cancer patients and healthy volunteers were found. It has been presented before that immunogenicity and seroconversion after the third booster dose are not impaired in solid cancer patients and that the control cohort achieves numerical, but not necessarily significantly higher anti-SARS-CoV-2 S1 IgG Ab levels [29,34,36].

One of the most important aspects of our study is the realization that none of our vaccinated patients with at least two doses of vaccine died because of the SARS-CoV-2 infection. Most patients had a mild disease course, and only two reported having a moderate disease course and required additional antiviral medication or synthetic antibodies. Additionally, no severe adverse events were reported after receiving the third booster dose of the vaccine, although adverse events were not followed as strictly as after the primary course of vaccination, where the study team actively contacted each patient after the vaccination dose was received. 

With the emergence of new variants of concern (VOC) such as Delta and Omicron, which have different characteristics of contagiousness, it is important to build the immune system defense of cancer patients as impeccable as possible for them to be able to proceed with the required treatment without the constant fear of lethal infection [20]. In this view, it has been shown that patients with cancer receiving the booster third dose of the vaccine display a significantly greater neutralizing capacity against the Omicron variant compared to those only receiving two doses of mRNA vaccines. With the new vaccine options, it is crucial to think of special populations, such as cancer patients, in advance and how such vaccines could be applied. [37] Additionally, neutralization breadth against different VOC correlates significantly with anti-SARS-CoV-2 S1 IgG Ab levels [24,28]. Surely, anti-SARS-CoV-2 S1 IgG Ab levels are easy to assess and reproduce and have been shown to be a valid surrogate for virus neutralization breadth [24]. After the initiative to harmonize anti-SARS-CoV-2 S1 IgG Ab levels and report them in BAU/mL, the results are now comparable and clear [20]. Although the shortcoming of this study is both a smaller number of patients included and not being able to assess T cell immunity or neutralization capacity of VOC being active at that moment, the strong side of it is the prospective design, preplanned time points for obtaining blood samples, and one central laboratory for Ab level assessment, which reduces the chances of differences in methodology and execution of the assay. 

## 5. Conclusions

In conclusion, this study, which is similar to others, has shown the positive experience of using the mRNA COVID-19 vaccines in patients treated for solid cancers, which produce both sufficient anti-SARS-CoV-2 S1 IgG Ab levels and protection against severe disease courses in the case of an infection. Hopefully, the COVID-19 pandemic is now a recent ordeal from which we must derive the best deductions possible. One of them must be to include cancer patients in future prospective trials when an issue of such importance as a vaccine against global pandemics comes into question. The other is the recognition that solid cancer patients derive similar, if not equal, benefits as their healthy counterparts from mRNA vaccines, which are both safe and highly effective in this special population. 

## Figures and Tables

**Table 1:** Demographic and clinical characteristics of included patients.

	N = 66
Age in years, median (range)	64 (43–83)
Sex, n (%)	
- Male	27 (41%)
- Female	39 (59%)
Cancer type, n (%)	
NSCLC ^†^	53 (80%)
- Breast cancer	7 (11%)
- Genitourinary cancer	3 (5%)
- Gastrointestinal cancer	2 (3%)
- Ovarian cancer	1 (1%)
Stage, n (%)	
- Limited	15 (23%)
- Locoregionaly advanced	5 (7%)
- Metastatic	46 (70%)
Anticancer therapy, n (%)	
- Chemotherapy	13 (20%)
- Immune checkpoint inhibitors	26 (40%)
- Targeted therapy	27 (40%)
Positive SARS-CoV-2 IgG antibodies prior to vaccination, n (%) ^‡^	
- No	57 (86%)
- Yes	9 (14%)
Type of primary vaccination received, n (%)	
- mRNA-based BNT162b2 (Pfizer/BioNTech)	60 (91%)
- mRNA-based mRNA-1273 (Moderna)	2 (3%)
- vector-based vaccine AZD1222 (AstraZeneca)	4 (6%)
Type of booster third-dose vaccination received, n (%)	
- mRNA-based BNT162b2 (Pfizer/BioNTech)	64 (97%)
- mRNA-based mRNA-1273 (Moderna)	2 (3%)
Median time between primary course of vaccination and third booster dose received, n (range)	216 days (153–300)

Legend: ^†^ NSCLC—non-small cell lung cancer; ^‡^ SARS-CoV-2 status was positive at baseline if the patient had clinical or virological evidence of COVID-19 illness either by positive patient history or positive RT-PCR test (reverse transcriptase–polymerase chain reaction); patients were considered to have positive SARS-CoV-2 IgG antibodies if the level of anti-SARS-CoV-2 S1 IgG was above the threshold of 40 BAU/mL.

## Data Availability

The data supporting the findings of this study are available within the manuscript. Any additional supporting data beyond the contained data are available from the corresponding author upon reasonable request.
